# Supplementary material for: Application of a Polysaccharide Purification Instrument—The Preparation and Characterization of Soybean Soluble Polysaccharide
Source: Polymers (Basel). 2025 Feb 12;17(4):480. doi: 10.3390/polym17040480 (PMC11859102; doi:10.3390/polym17040480)

Supplementary Materials

Application of a Polysaccharide Purification Instrument —The Preraration and Characterization of Soybean Soluble Polysaccharide

Xuhui Zhuang, Hongjuan Chen, Xiaohong Luo, Wei Han*, and Yongtan Yang*

Academy of National Food and Strategic Reserves Administration, Beijing 100037, P. R. China ; zxh@ags.ac.cn
* Correspondence: hw@ags.ac.cn; yyt@ags.ac.cn

Citation: To be added by editorial staff during production.

Academic Editor: Firstname Lastname

Received: date
Revised: date
Accepted: date
Published: date

Copyright: © 2024 by the authors. Submitted for possible open access publication under the terms and conditions of the Creative Commons Attribution (CC BY) license (https://creativecommons.org/licenses/by/4.0/).

Figure S1: FT-IR spectrum of SSPS-P

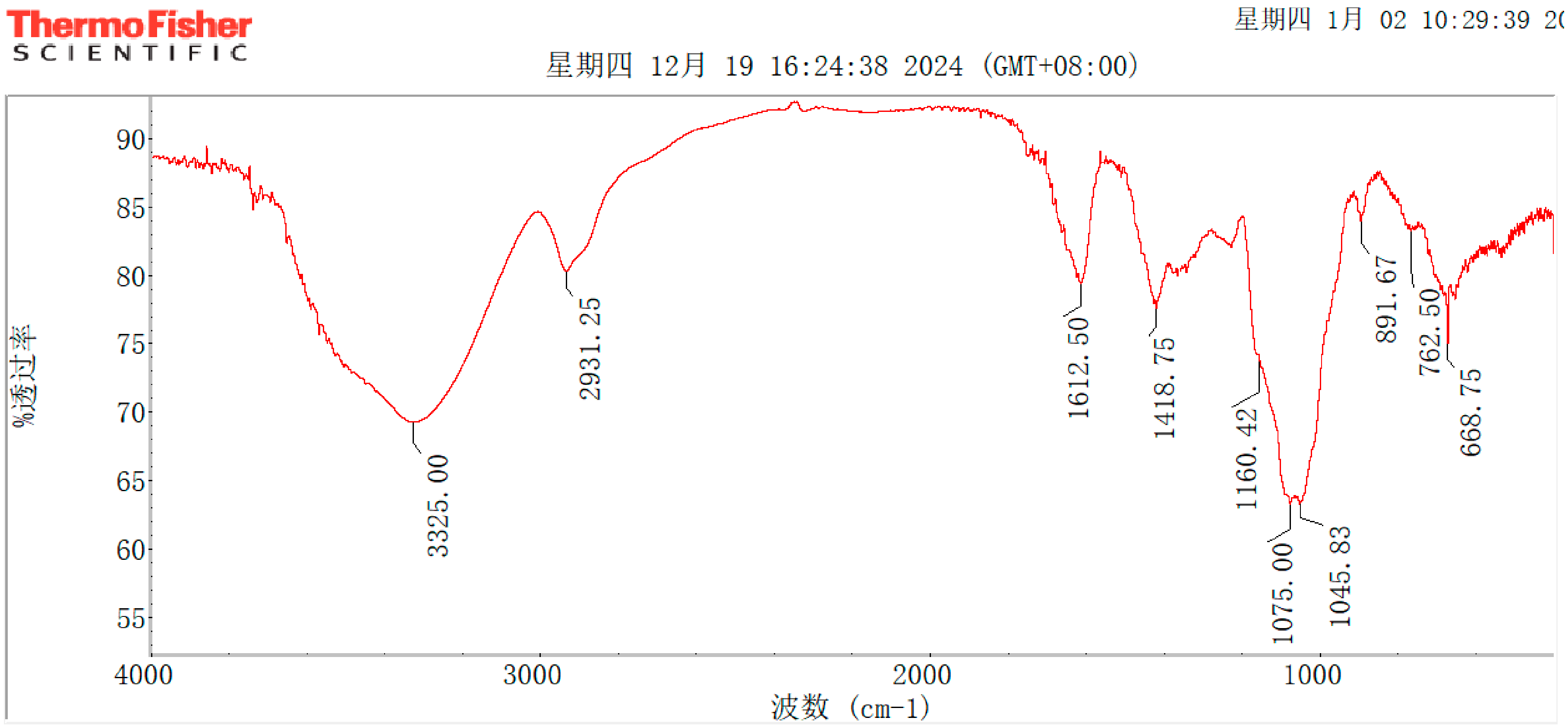

Figure S2: ¹H-NMR spectrum of SSPS-P

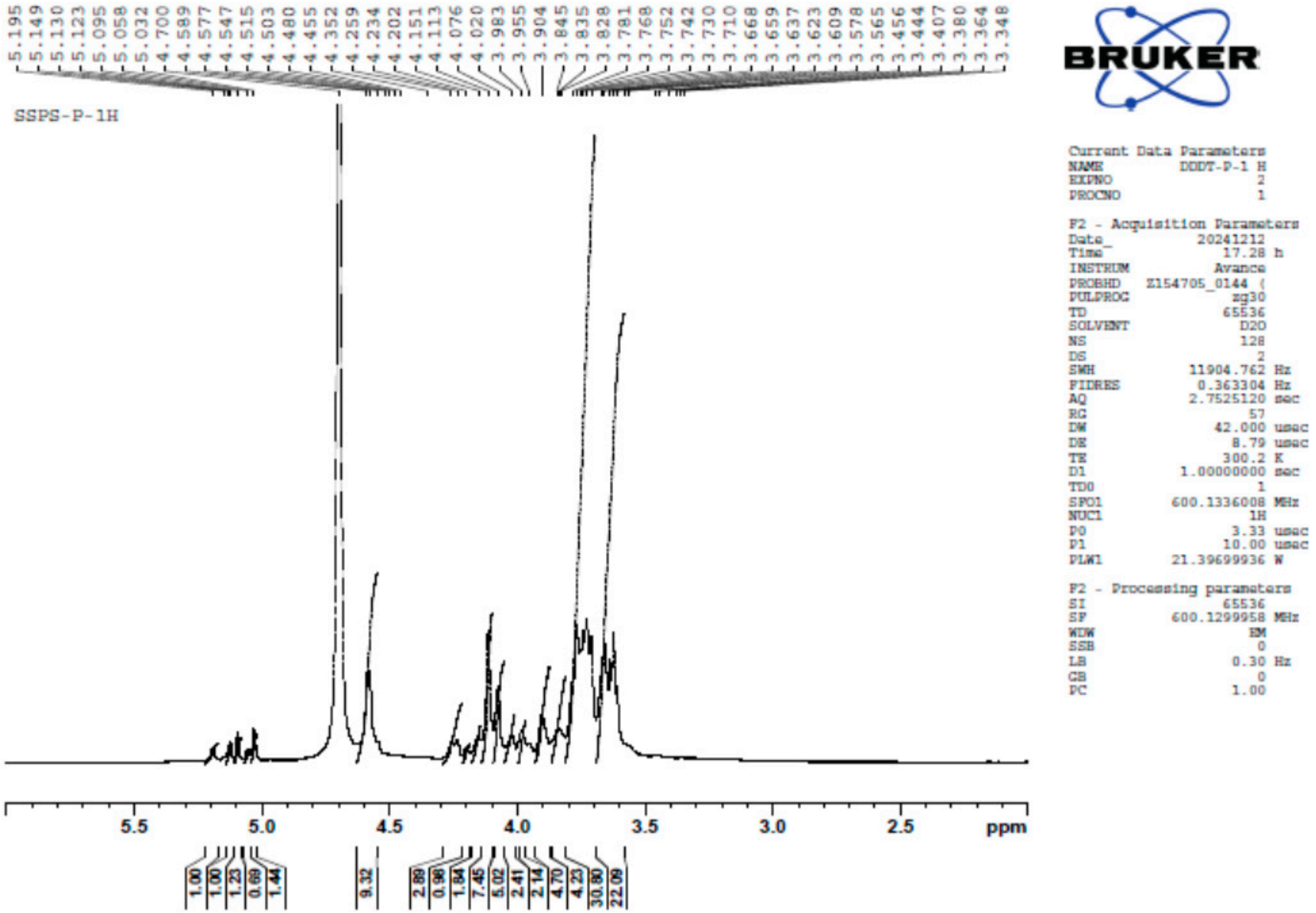

Figure S3: ^{13}C-NMR spectrum of SSPS-P

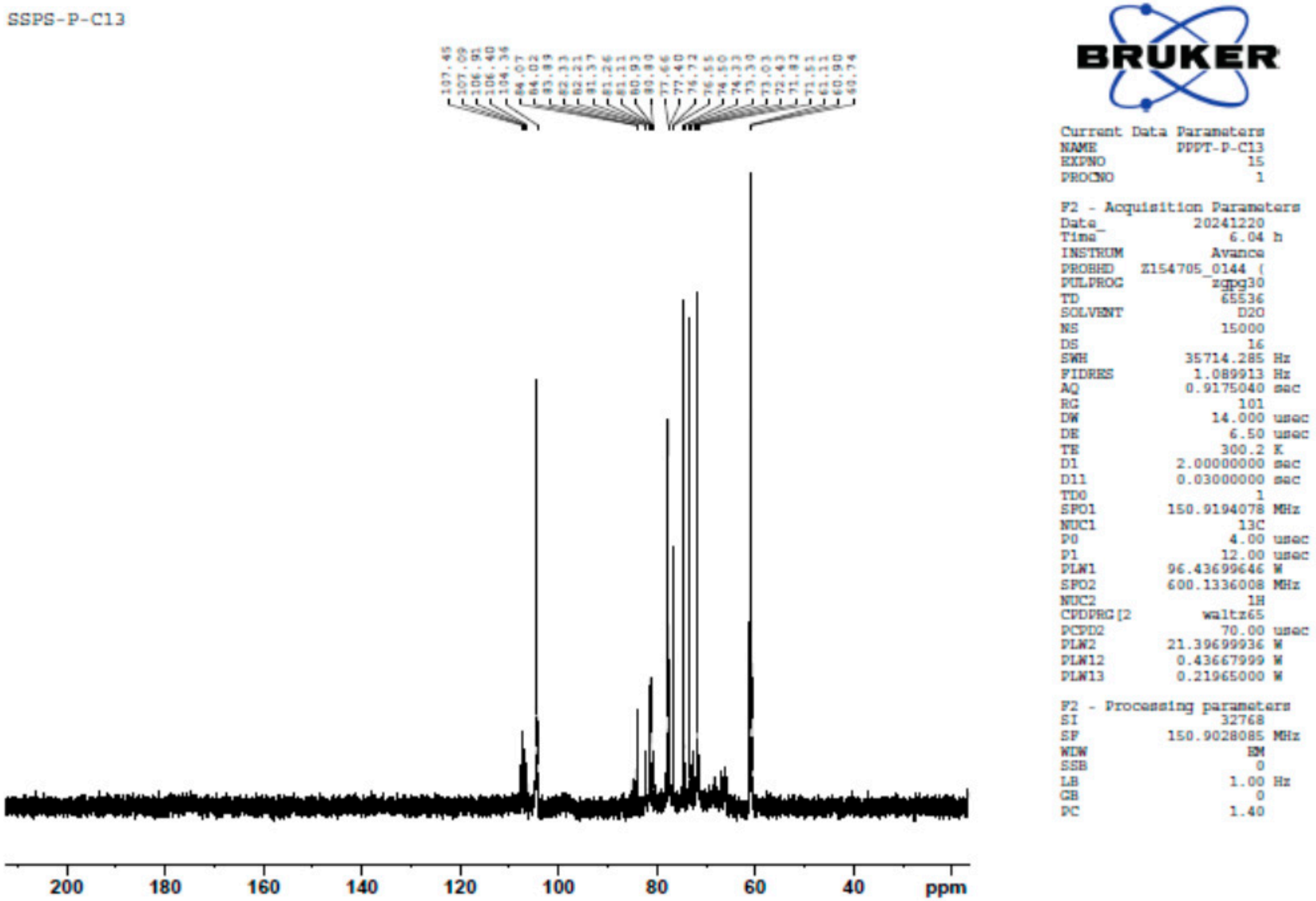

Figure S4: 135 DEPT spectrum of SSPS-P

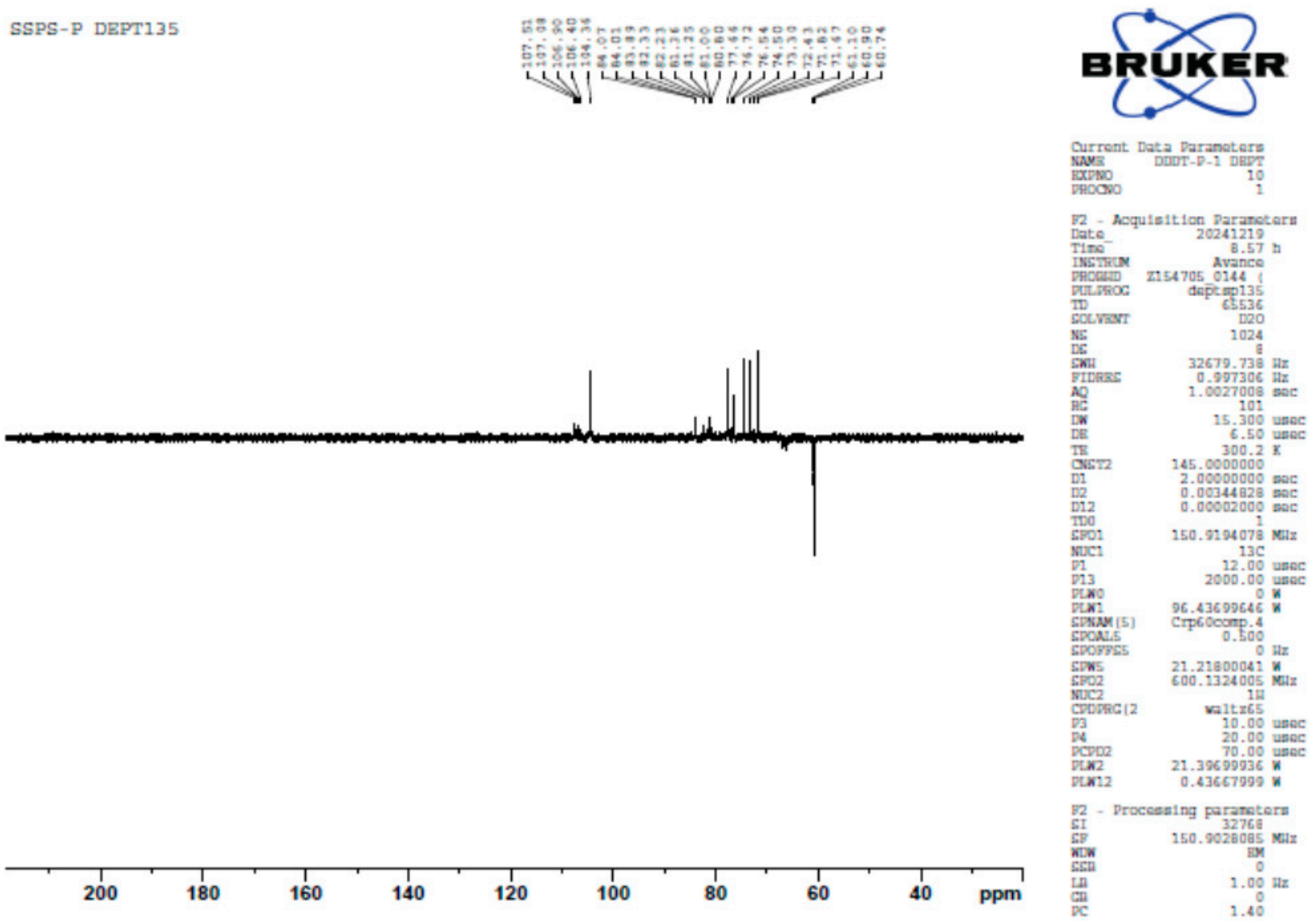

Figure S5: TOCSY spectrum of SSPS-P

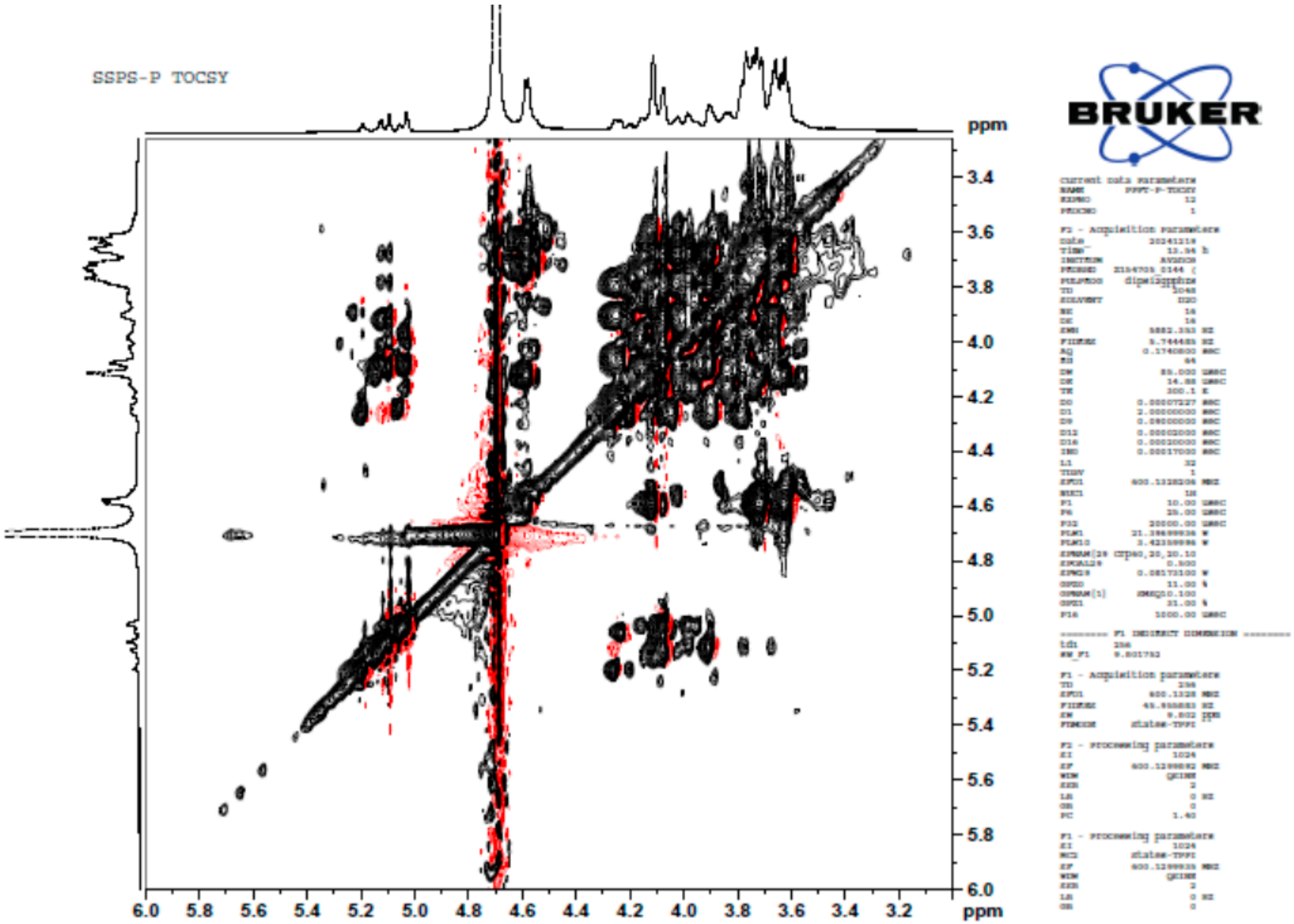

Figure S6: HSQC spectrum of SSPS-P

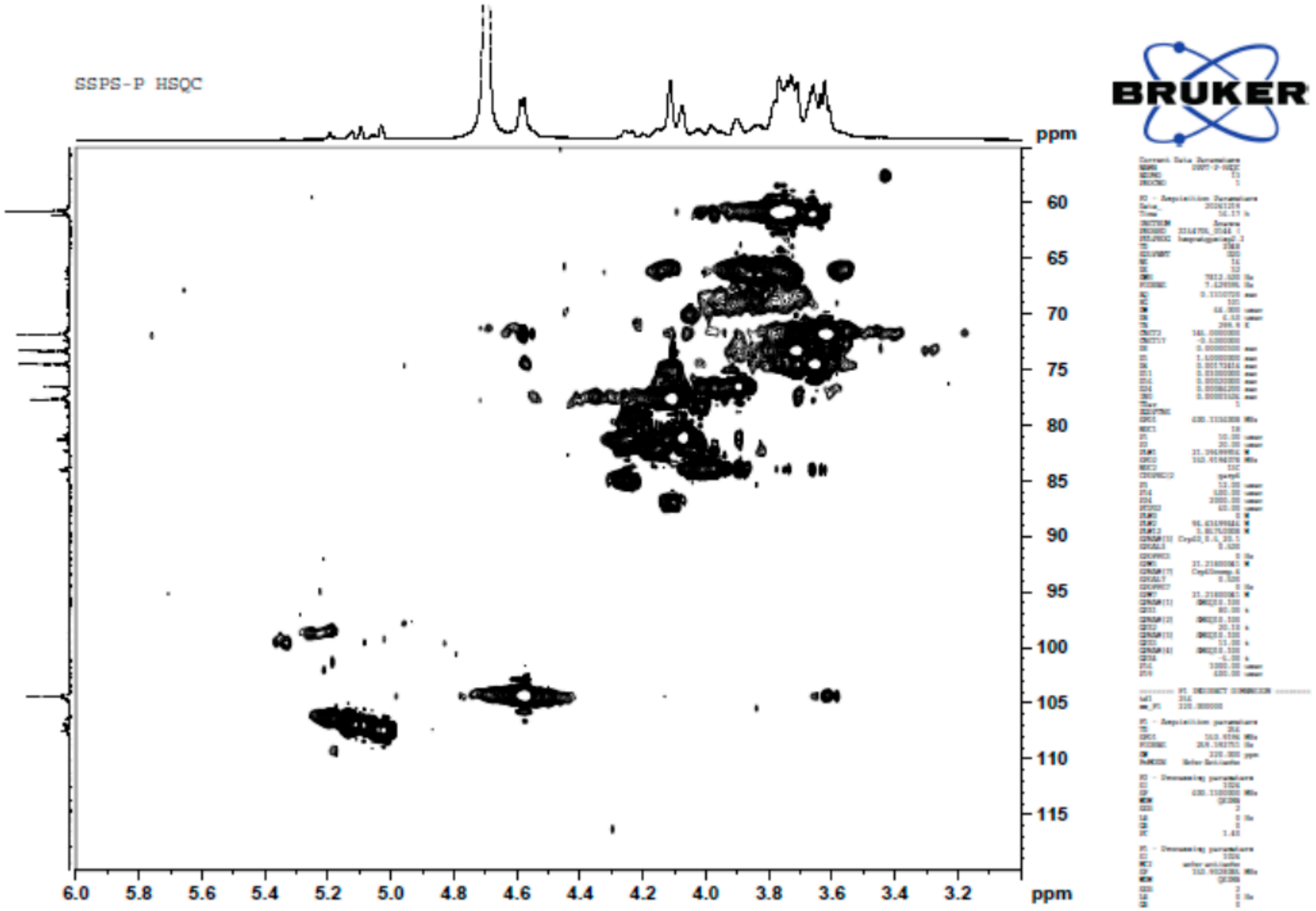

Figure S7: ^{1}H-^{1}H COSY spectrum of SSPS-P

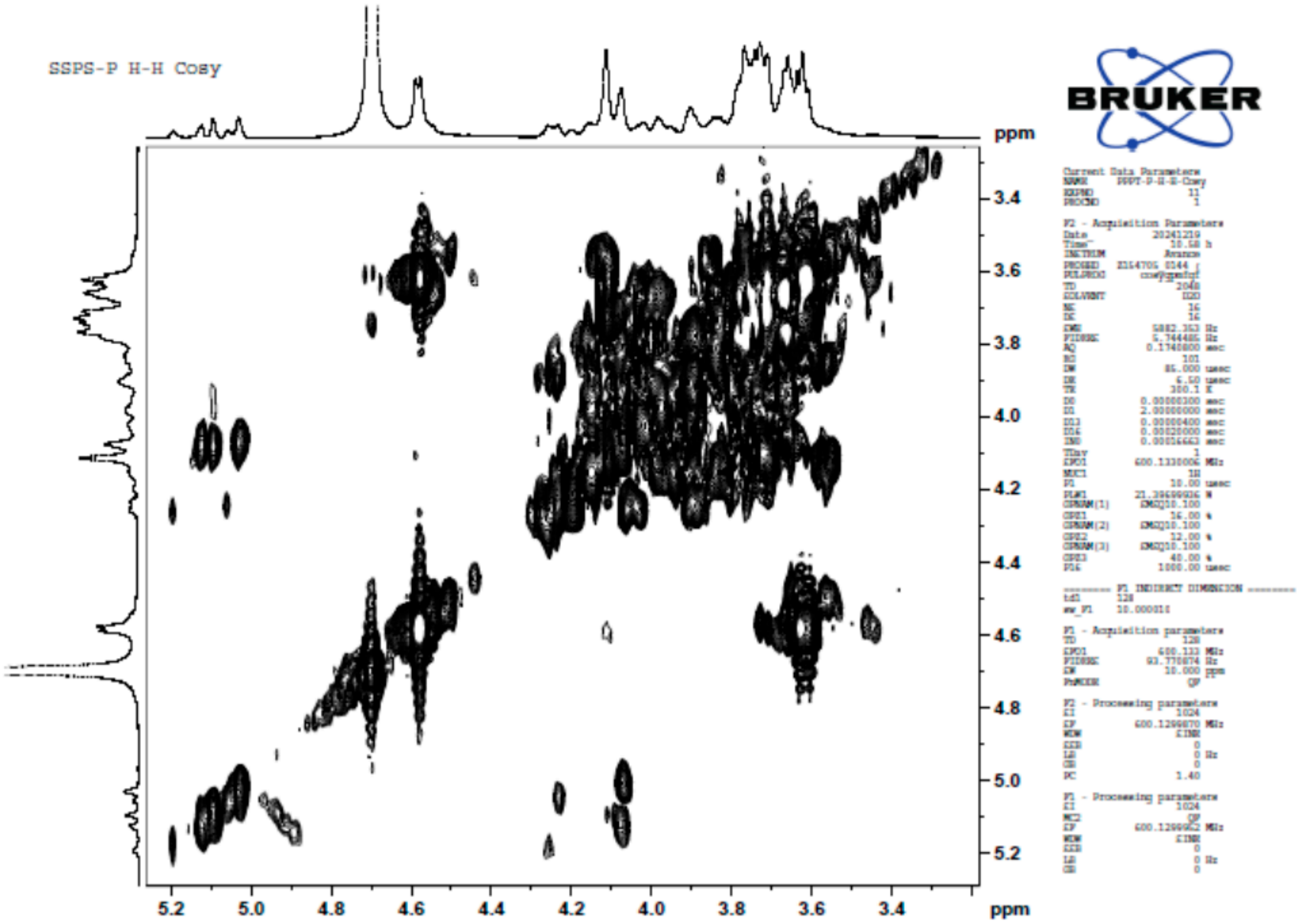

Figure S8: HMBC spectrum of SSPS-P

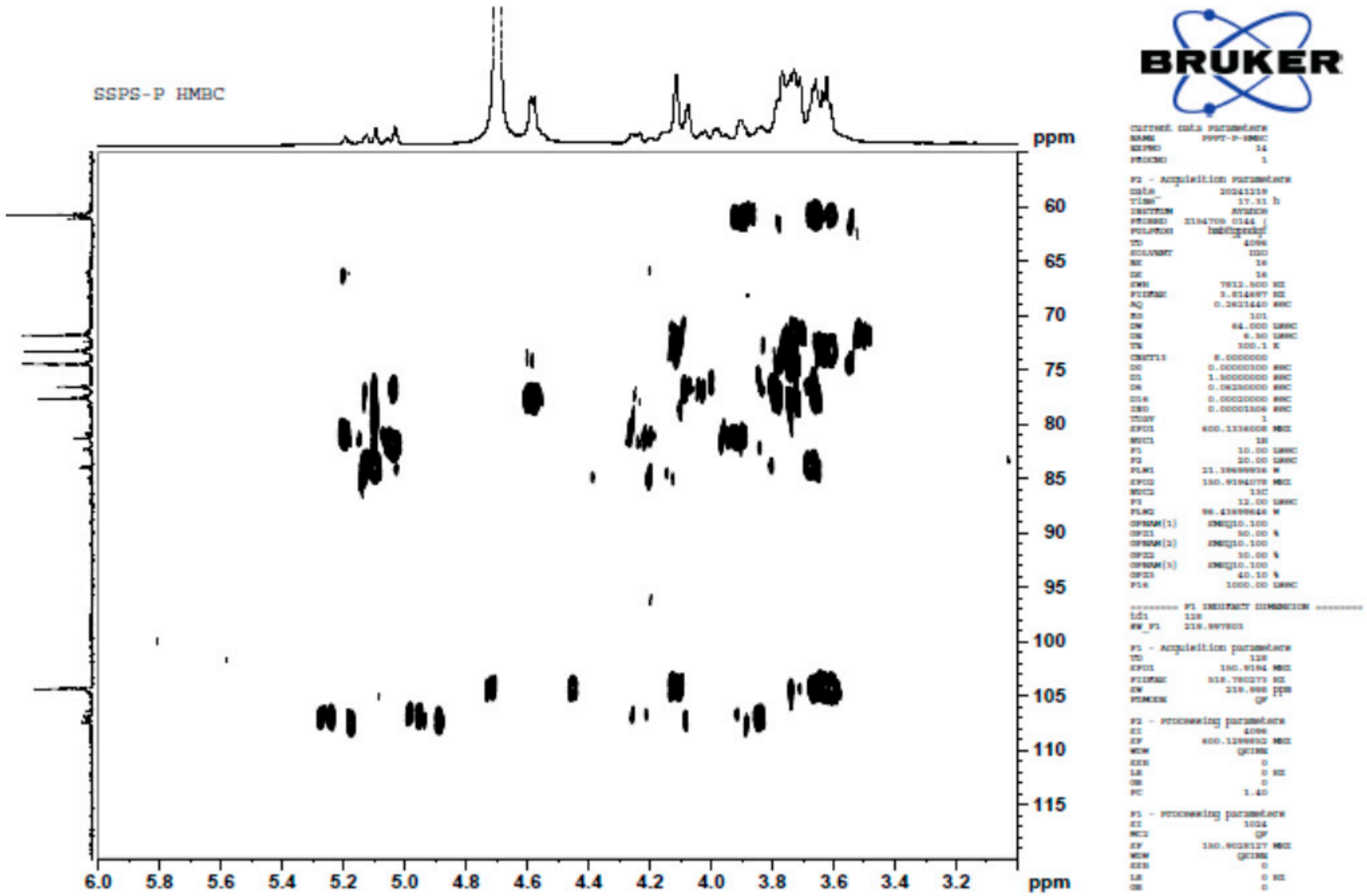

Supplement: Supplementary file 1 [file polymers-17-00480-s001.zip › polymers-3436816-supplementary.pdf]
